# No gains for bigger brains: Functional and neuroanatomical consequences of relative brain size in a parasitic wasp

**DOI:** 10.1111/jeb.13450

**Published:** 2019-04-13

**Authors:** Emma van der Woude, Jitte Groothuis, Hans M. Smid

**Affiliations:** ^1^ Laboratory of Entomology Wageningen University Wageningen The Netherlands

**Keywords:** appetitive olfactory conditioning, bidirectional artificial selection, brain scaling, brain size, longevity, *Nasonia vitripennis*, parasitic wasp

## Abstract

Heritable genetic variation in relative brain size can underlie the relationship between brain performance and the relative size of the brain. We used bidirectional artificial selection to study the consequences of genetic variation in relative brain size on brain morphology, cognition and longevity in *Nasonia vitripennis* parasitoid wasps. Our results show a robust change in relative brain size after 26 generations of selection and six generations of relaxation. Total average neuropil volume of the brain was 16% larger in wasps selected for relatively large brains than in wasps selected for relatively small brains, whereas the body length of the large‐brained wasps was smaller. Furthermore, the relative volume of the antennal lobes was larger in wasps with relatively large brains. Relative brain size did not influence olfactory memory retention, whereas wasps that were selected for larger relative brain size had a shorter longevity, which was even further reduced after a learning experience. These effects of genetic variation on neuropil composition and memory retention are different from previously described effects of phenotypic plasticity in absolute brain size. In conclusion, having relatively large brains may be costly for *N. vitripennis*, whereas no cognitive benefits were recorded.

## 1 INTRODUCTION

Brain size is linked to brain performance through the number of neurons and their connectivity (Chittka & Niven, 2009; Striedter, 2005). Variation in brain size can therefore underlie differences in cognitive abilities (Dicke & Roth, 2016). The effect of brain size on cognition depends on body size, since smaller animals do not require the same absolute brain size as larger animals. However, relative brain size does increase with decreasing body size, a phenomenon described by Haller's rule (Rensch, 1948, 1956). The relationship between brain size and body size follows a power law function. In the case of a negative allometry that is described by Haller's rule, the scaling coefficient of this power law function is smaller than 1, both for interspecific (e.g. Harvey & Krebs, 1990; Isler et al., 2008; Pagel & Harvey, 1988; Wehner, Fukushi, & Isler, 2007) and intraspecific (e.g. Riveros & Gronenberg, 2010; Seid, Castillo, & Wcislo, 2011; Wehner et al., 2007) comparisons.

Certain complex behavioural adaptations, like foraging for a specific prey, may drive the evolution of enhanced cognitive abilities to optimize an animal's fitness. This may be realized by a relatively larger brain. However, the relatively high developmental and operating costs of brain tissue (Aiello & Wheeler, 1995; Mery & Kawecki, 2005) may incur negative effects on fitness and longevity when animals have a relatively larger brain. Such metabolic constraints may be more severe in populations that evolve under stringent dietary conditions than in populations that evolve under more permissible conditions. For small animals, the high metabolic costs of the relatively large brain put a stronger constraint on energy expenditure for brain development and operation compared to larger animals. To be able to adapt relative brain size to different ecological circumstances, phenotypic plasticity and genetic variation must be present. The latter aspect was studied recently in guppies (Kotrschal et al., 2013), using a bidirectional artificial selection regime. The resulting selected lines with either relatively large or small brains showed correlated effects on learning abilities (Kotrschal, Corral‐Lopez, Amcoff, & Kolm, 2015; Kotrschal et al., 2013), gut mass (Kotrschal et al., 2013), survival (Kotrschal, Buechel, et al., 2015), proactiveness (Kotrschal et al., 2014), sexual traits (Kotrschal, Corral‐Lopez, Zajitschek, et al., 2015) and the immune system (Kotrschal, Kolm, & Penn, 2016), reflecting the trade‐offs involved in developmental expenditure of resources in cognition vs somatic processes. The differences in relative brain size between large‐ and small‐brained guppies were caused by differences in the expression of only a single gene: angiopoietin‐1 (Chen et al., 2015).

Phenotypic plasticity can be regulated by genetically encoded developmental programmes (e.g. Lanet & Maurange, 2014). These determine how a single genotype morphologically responds to different developmental conditions, such as differences in nutritional levels, caste differentiation and sex determination. Natural genetic variation in the plasticity genes that facilitate these differential development programmes may predispose animals to optimize their development to match specific ecological circumstances, such as low food availability.

Parasitic wasps are ideal model animals to study these aspects of variation in relative brain size. These insects lay their eggs in other insects and develop inside their host insects. Exploiting different host species, with differences in host oviposition behaviour and host habitat, may require different foraging strategies and therefore different cognitive abilities of the parasitic wasps (Kruidhof et al., 2012; Smid & Vet, 2016; Smid et al., 2007), while also requiring adaptations to differences in host quality or size. For instance, the parasitic wasp *Nasonia vitripennis* lays its eggs in a wide variety of species of fly pupae, which can occur in different habitats, such as dung, corpses or bird nests. The adult size of these wasps varied, as established in the isogenic strain AsymCx so due to phenotypic plasticity alone, between 1.2 and 2.4 mm, which represents a factor 10 in dry body weight (Groothuis & Smid, 2017). The size depends on the quality and size of the host, but also on the level of scramble competition between larvae that develop within the same host. This phenotypic plasticity of body size has a remarkable effect on relative brain size in this small insect species. The wasps show diphasic brain–body size scaling with isometry in the range of smaller body sizes and negative allometry in the range of larger body sizes, possibly because they switch to a different developmental programmes (Groothuis & Smid, 2017). Large *N. vitripennis* show higher levels of olfactory and visual memory retention than small *N. vitripennis* (Van der Woude, Huigens, & Smid, 2018). This may be related to differences in relative neuropil volumes. In the smallest wasps, the mushroom bodies, known to be important for memory formation in other insects (Perry & Barron, 2012), were relatively smaller; on the other hand, the relative volume of the lateral horn, known to be involved in naive responses to olfactory cues (Parnas, Lin, Huetteroth, & Miesenbock, 2013; Strutz et al., 2014), had not changed. This may indicate that, when challenged with restricted resources, isogenic *N. vitripennis* are able to utilize different developmental programmes and develop differentially structured brains. In this example, the decrease in absolute and relative mushroom body volume may underlie their aforementioned lower memory performance.

Here, we used *N. vitripennis* to study the consequences of genetic variation in relative brain size using constant, low levels of scramble competition to minimize phenotypic effects on body size. This was done by means of a bidirectional artificial selection regime, using the ratio between head width and body length as proxy for relative brain size during the selection process. We used an outbred population of *N. vitripennis* that was specifically collected and maintained to preserve natural genetic variation (Van de Zande et al., 2014). We studied the effects of this selection regime on brain volume, brain structure, cognition and longevity. We expected that there is heritable variation in relative brain size under constant nutritional levels. We expected that (A) there is a positive correlation between relative brain size and memory performance, (B) relative neuropil volumes are affected by selection for relative brain size and (C) there is a negative correlation between relative brain size and longevity.

## 2 MATERIALS AND METHODS

### 2.1 Insects

We used female *N. vitripennis* Walker (Hymenoptera: Pteromalidae) of strain HVRx, which was specifically collected and maintained to preserve natural genetic variation (Van de Zande et al., 2014). The wasps were reared on *Calliphora vomitoria* pupae (obtained as maggots from Kreikamp B.V.) and kept in a climate cabinet at 20 ± 1°C with a 16:8 L:D cycle. The generation time was ca. 3 weeks.

### 2.2 Selection regime

To initiate the selection lines, 200 mated female *N. vitripennis* were sedated with CO_2_. Body length and head width of these wasps were measured using a dissection microscope with ocular micrometre. The ratio between head width and body length was calculated and used as proxy for relative brain size, in order to enable the selection process on living wasps. Previous results in *N. vitripennis* (Groothuis & Smid, 2017) and in another parasitoid species*, T. evanescens* (van der Woude, Smid, Chittka, & Huigens, 2013), showed that the brain lies tightly adjacent to the head capsule with very little additional space for other tissues, and in the latter species with a correlation (*R* ^2^) between head capsule volume and brain volume of 0.98. Note that this proxy was not used for the final assessment of brain size (see below). The 30 wasps with the largest ratio were randomly distributed over three rearing vials in groups of 10 wasps, to initiate three selection lines for large heads (defined as Large (L)). Similar procedures were used to initiate three selection lines for small heads (defined as Small (S)), using the 30 wasps with the smallest ratio.

Another set of 30 wasps were randomly selected from the starting population and used to initiate three control lines (defined as Control (C)) to control for the effect of selection on inbreeding. This resulted in three replicate lines per selection regime: large L1, L2, L3, small S1, S2, S3 and control C1, C2, C3. Each rearing vial contained 20 *C. vomitoria* pupae and a drop of honey.

In every subsequent generation, 50 mated female wasps per S and L line were sedated and measured as described above. The 10 wasps with the largest (for L) and smallest (for S) ratios between head width and body length were used to initiate the next generation. For the C lines, 10 randomly chosen females were used, without measurements. These selection procedures were repeated for 25 generations. After the 25th generation, selection was relaxed, with the exception of generations 30, 33 and 40.

### 2.3 Neuropil staining and relative neuropil measurements

Per replicate line, 12 randomly selected female wasps (i.e. 108 in total) from generation 33 were sedated on ice, after which they were decapitated in ice‐cold phosphate‐buffered saline (PBS, Oxoid, Dulbecco “A” tablets). The brains were removed using sharpened tweezers (Dumont #5; Sigma), placed in phosphate‐buffered (0.1 M) 4% formaldehyde solution (pH 7.2) and fixed for 2.5 hr at room temperature. After fixation, the brains were rinsed in PBS 6 times for 5 min and treated with 5 mg/ml collagenase (Sigma) in PBS for 1 hr at RT. Following rinsing in PBS containing 0.5% Triton X‐100 (PBS‐T) 4 times for 5 min, brains were incubated for 1 hr in blocking buffer, PBS‐T containing 10% normal goat serum (PBS‐T‐NGS; Dako). Incubation in primary antibody, against the presynaptic Bruchpilot protein (Wagh et al., 2006) (mouse anti‐Bruchpilot concentrate, NC82‐c; Developmental Studies Hybridoma Bank, University of Iowa, Iowa City, IA, USA; Cat. no. nc82, RRID:AB_528108) diluted 1:250 in PBS‐T‐NGS, was overnight at RT, followed by six times 20 min rinsing in PBS‐T and 4‐h incubation at RT in secondary antibody, 1:100 rabbit anti‐mouse (Dako) in PBS‐T‐NGS. After another 6 times 20 min rinse in PBS‐T, the brains were incubated overnight at 4°C in tertiary antibody, 1:200 Alexa Fluor^®^ 488‐conjugated goat anti‐rabbit (Invitrogen) and 1:250 propidium iodide (Sigma‐Aldrich) in PBS‐T‐NGS. Subsequent steps were performed in the dark as much as possible. Brains were dehydrated through a series of increasing EtOH dilutions (30%, 50%, 70%, 80%, 90%, 96%, 2 × 100%), degreased via a 50/50 EtOH/xylene step and kept in xylene until mounting. Brains were mounted in DPX (Sigma) between a glass microscope slide, fitted with two stacked strips of double‐sided adhesive tape (Henzo) as spacer, and a 18 mm × 18 mm #1 cover slip.

Whole‐mount Z‐stacks were acquired using a Zeiss LSM 510 confocal microscope equipped with a Plan‐Neofluar 25 × /0.8 oil immersion objective. Alexa Fluor^®^ 488 and PI were excited using the Ar‐488 nm line and captured with 505–550 nm BP and 560 nm LP filters, respectively. Images were obtained at 512 × 512 px with a 0.7 × digital zoom and a step size of 2 μm, resulting in a final voxel calibration of 1.018 × 1.018 × 2 μm. As the refractive indices of immersion and mounting medium match, no z‐correction was required. Depending on the size and orientation of a scanned brain, 1 to 3 stacks were acquired and later combined with the Stitching plugin (Preibisch, Saalfeld, & Tomancak, 2009) in FIJI (Schindelin et al., 2012). Due to the fragile nature of *Nasonia* brains (Haverkamp & Smid, 2014), we inspected the obtained stacks for integrity of all neuropils and selected 3 of 12 brains of every line for analysis (resulting in 9 brains per treatment and 27 brains for the entire experiment). Due to its tight connection with the eye, the optic lobe lamina is often damaged during dissection. Therefore, it was not included in this analysis.

Neuropil segmentation was performed in Amira 5.4.2 (Visage Imaging). The nc82 channel was used to assign 11 unique labels to the neuropil in the Segmentation Editor, see Figure 3 in the main text. Each neuropil was manually labelled each 1–3 slices, after which the interpolate option was used. Manual correction was performed to ensure correct labelling of each slice. Neuropil volumes were calculated by the MaterialStatistics module and saved as .csv file for collection and calculation of relative volume in an MS Excel spreadsheet. In one case, a brain turned out to have previously unnoticed damage to one of the calyces. For this brain, the duplicated volume of the undamaged calyx was used to calculate the total calyx volume. Relative neuropil volume was calculated as the percentage of the total neuropil volume.

### 2.4 Memory retention

Olfactory memory retention of the selection lines was tested in generation 33. We used single classical olfactory conditioning trials, with a T‐maze for testing of memory retention as described before (Hoedjes, Smid, Vet, & Werren, 2014; Van der Woude et al., 2018). The wasps were 1–2 days old and kept on water and honey until use in the conditioning trials. Groups of approximately 60 wasps were distributed over a Petri dish (8.5 cm diameter). Here, the wasps obtained an oviposition experience (unconditioned stimulus, US) while experiencing an odour (conditioned stimulus, CS): the CS+ phase. The rewarding unconditioned stimulus consisted of 40 *C. vomitoria* pupae. The conditioned stimulus was 5 μl of either Royal Brand Bourbon Vanilla extract or Natural Chocolate extract (Nielsen‐Massey Vanillas Intl.), pipetted on small squares of filter paper. The wasps were allowed to drill in the pupae for 1 hr, while experiencing the odour of the CS+. Wasps that were not drilling in the pupae were removed after 15 min. After 1 hr, the wasps were removed from the pupae with an aspirator and placed in a clean petri dish for a neutral resting phase of 15 min. Next, the wasps experienced 5 μl of the second of the two odours in the absence of hosts: the CS‐ phase. This phase lasted for another 15 min. After this phase, the wasps were collected in clean vials and stored with water and honey until use in the memory retention tests.

The conditioning trials were performed in a reciprocal manner: one group of every line was conditioned using vanilla as CS+ and chocolate as CS‐, and another group was conditioned using chocolate as CS+ and vanilla as CS−. Four groups per replicate line were conditioned on chocolate, and four groups per replicate line were conditioned on vanilla.

For memory testing, one side of the T‐maze contained a glass capillary (ID 1.3 mm, Stuart SMP1/4; Bibby Scientific) filled with vanilla extract, and the other side contained chocolate extract. Charcoal filtered, moisturized air (60–70% relative humidity) flowed past the odour capillaries at 100 ml/min per side. Wasps were inserted in the T‐maze in groups of approximately 15 wasps, resulting in three measurements per conditioned group. Memory of each wasp was tested 1, 3 and 5 days after the conditioning trials. After 5 min, the number of wasps on the vanilla and chocolate side was recorded.

### 2.5 Longevity

Longevity was studied in generation 40. Wasps of each replicate selection line were used either naively or after an olfactory conditioning trial (as described above). Each replicate line was analysed with two groups of naive and two groups of conditioned wasps, each group containing 30 wasps. These groups were placed in clean rearing tubes with unlimited access to water and honey and kept in a climate cabinet at 25°C. The tubes were refreshed weekly. Every 2 days, the number of dead wasps was counted.

### 2.6 Statistical analyses

Response to selection was analysed using a linear mixed model with the ratio between head width and body length as dependent variable. Selection regime (L or S), generation and the interaction between these two were used as fixed factors. Replicate number was used as a random factor. Deviance of model terms was analysed using type II Wald chi‐square tests. Differences in the head–body size ratio between the selection regimes (L or S) within the generations were determined with chi‐square pairwise comparisons. Similar linear mixed models were used to test the selection's effect on body length and head width, using, respectively, the natural logarithm of body length or head width as dependent variable.

Ordinary linear regression on head width and mean‐centred body length was used to study whether the difference in head–body size ratio between the selected lines can be explained by allometric brain scaling in combination with differences in body size. Head width was used as dependent variable, and body length and selection regime (L, C or S) as fixed factors. Body lengths were mean‐centred by subtraction of the average body length of all wasps in that generation. This ensured that differences in the intercept reflect differences in head–body ratio between the selected lines, as head width is compared at mean‐centred body length (Egset, Bolstad, Rosenqvist, Endler, & Pelabon, 2011; Tsuboi et al., 2016). If there are still differences in head–body ratio at mean‐centred body length, these are not caused by allometric brain scaling resulting from the difference in body size between the lines. ANOVA comparisons were used to test for differences in slope and intercept between the lines. We used this method to analyse wasps separately for generation 26, 33 and 40.

We calculated realized heritability after 25 generations of selection. We used the ratio between the cumulative selection response and the cumulative selection differential, following the method for divergent selection described by Walsh and Lynch (2018). The cumulative selection response was defined as the difference in mean head–body ratio between L and S in generation 26. The cumulative selection differential was defined as the cumulative difference in selection differentials (mean head–body ratio of the selected group subtracted from the mean of that whole population) between L and S of 25 generations. The value for realized heritability was duplicated to correct for selection on only females, instead of on both parents.

Differences in neuropil volumes were analysed in generation 33. Neuropil volumes were compared with a linear mixed model. We used the absolute total neuropil volume or relative volume per neuropil as dependent variables, with selection regime as fixed factor and line as random factor. As we compared multiple relative neuropil volumes, we corrected the *p*‐values for multiple comparisons with the Holm–Bonferroni method (*m *=* *11) (Holm, 1979) in MS Excel. Neuropils with significant effects of selection regime on relative volume were further analysed with chi‐square pairwise comparisons to test for significant differences between the selection regimes.

Differences in memory retention abilities were analysed in generation 33. Memory retention was expressed as a performance index (PI): the difference in preference between reciprocally trained groups. This PI is calculated by subtracting the fraction of wasps that chose the odour of their CS‐ from the fraction of wasps in the reciprocal group, which chose that same odour but received it as their CS+. Values of PIs were calculated from estimated response means that were obtained from generalized linear mixed models (GLMMs) with logit link function and binomial distribution. The dependent variable was the number of wasps that chose chocolate with the total number of wasps making a choice as denominator. Fixed effects included the odour of CS+, time after conditioning, selection line and the interactions between these effects. Random effects were included to correct for date of conditioning, selection line repeat and reciprocal conditioning pair. The presence of memory was tested with chi‐square pairwise comparisons, which test for the effect of CS+ on the preference for the conditioned stimuli. Similar tests were used to analyse differences in memory retention between the different lines. Response rates of the memory retention tests were determined by a GLMM that used the fraction of wasps making a choice out of the total number of wasps inserted as dependent variable, and selection regime and time after conditioning as fixed factors. Differences in response rate between the lines and times were determined with chi‐square pairwise comparisons.

Longevity was analysed in generation 40. We used a two‐way ANOVA that tested for the effect of selection regime, conditioning and the interaction between these terms using time till death as dependent variable. This was followed by Tukey HSD post hoc tests to analyse differences in longevity between selected lines and to test for an effect of conditioning on longevity within selected lines.

Statistical analyses were performed in R version 3.1.0 using packages lme4 (Bates, Maechler, Bolker, & Walker, 2014), phia (De Rosario‐Martinez, 2013) and lsmeans (Lenth & Hervé, 2014).

## 3 RESULTS

### 3.1 Selection regime

**Figure 1:**
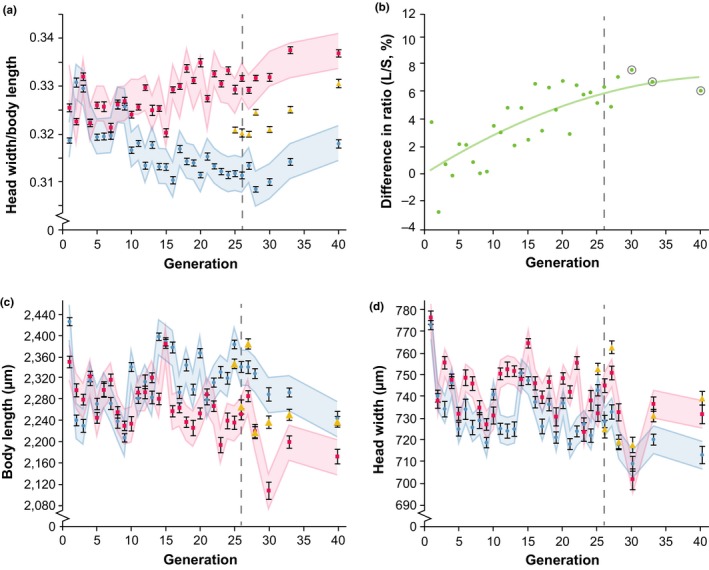
Relative brain size responds to bidirectional selection. Data points depict means over all individuals of all lines in a selection regime. Magenta squares: wasps selected for relatively large brains (L lines); blue circles: wasps selected for relatively small brains (S lines); yellow triangles: wasps of the control treatment (C lines). Dashed vertical lines in panels a‐d show the start of relaxation of the selection regime, and grey circles in panel B show generations used for additional selection. Linear mixed model predictions were used to calculate confidence intervals. (a) Relative brain size is shown as the mean ± SE of the head–body size ratio for all wasps of a certain selection regime. (b) Difference in the head–body size ratio between the L and S lines increases with each selected generation. Regression formula: y = −0.0035x^2^ + 0.317x, *R* ^2^=0.651. (c) Absolute body length (mean ± SE) and (d) absolute head width (mean ± SE) both respond to selection. Note that L wasps have shorter bodies than S (panel c), but wider heads (panel D)

There was a significant effect of the selection regime on the head–body size ratio ($\chi_{1}^{2}$ = 4496.16, *p *<* *0.001; Figure 1a). After generation 25 (the last generation undergoing selection), the difference between wasps of the large (L) and small (S) lines in head–body size ratio was 6.30% (Figure 1b, $\chi_{1}^{2}$ = 257.118, *p *<* *0.001). In generation 33, we assessed brain morphology and memory retention (discussed below); in this generation, the difference in head–body size ratio was 6.67% ($\chi_{1}^{2}$ = 440.762, *p *<* *0.001). We assessed longevity in generation 40, and here the difference in ratio was 6.03% ($\chi_{1}^{2}$ = 368.943, *p *<* *0.001). On average, the final differences in ratio were 6.41% in generations 26 to 40 (Figure 1b). Generation number significantly affected head–body size ratio ($\chi_{30}^{2}$  = 898.47, *p *<* *0.001), as did the interactions between selection regime and generation ($\chi_{30}^{2}$ = 1996.18, *p *<* *0.001). Realized heritability (*h* ^2^) of the ratio was 0.067 in generation 26.

Selection regime (for small vs. large head–body size ratio) had a significant effect on body length (Figure 1c) ($\chi_{1}^{2}$ = 322.437, *p *<* *0.001; Figure 1c). Body length was also affected by generation ($\chi_{30}^{2}$ = 888.169, *p *<* *0.001), and the interaction between selection and generation was significant ($\chi_{30}^{2}$ = 537.050, *p *<* *0.001). Selection regime also affected head width (Figure 1d) ($\chi_{1}^{2}$ = 202.113, *p *<* *0.001; Figure 1d), as did generation ($\chi_{30}^{2}$ = 864.363, *p *<* *0.001), and the interaction between selection and generation was significant ($\chi_{30}^{2}$ = 191.226, *p *<* *0.001).

**Figure 2:**
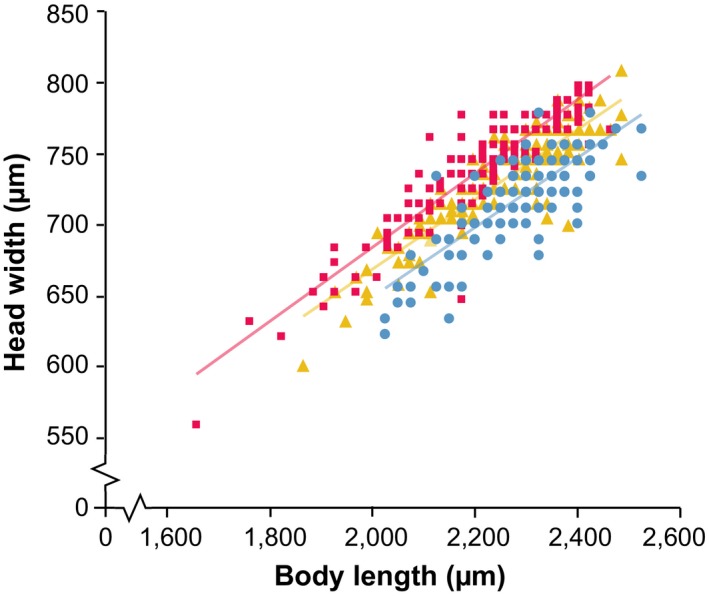
Head width and body length of individual wasps selected for relatively large (magenta squares) and small (blue circles) head–body ratio, and unselected control lines (yellow triangles). Data are shown for generation 33, which is the same generation used to study neuropil composition and memory performance. Regression analysis was performed on mean‐centred body lengths, which ensured that differences in the intercept reflected differences in head–body ratio. This revealed differences in the intercepts, but not in the slopes. Similar results for generation 26 and 40 are shown in Figure S2

Figure 2 shows the relationship between head width and body length in wasps of the three lines in generation 33. Linear regression on head width and mean‐centred body length revealed significant differences between the lines in generation 33 in intercept (L: 749.048, C: 730.396, S: 709.134; *F*_2,444_ = 36.466, *p *<* *0.001), but not in slope (L: 0.260, C: 0.245, S: 0.244; *F*_2,444_ = 0.670, *p* = 0.512). Similar results were found for wasps of generations 26 and 40 (see Figure S2). This shows that wasps of the L, C and S lines differ in head width independent of the body size effects due to selection. The effect on head–body size ratio is, therefore, not caused by allometric brain scaling resulting from the difference in body size between the lines. Body lengths, head widths and ratios between head width and body length for all generations are shown in Table S1.

### 3.2 Brain morphology

**Figure 3:**
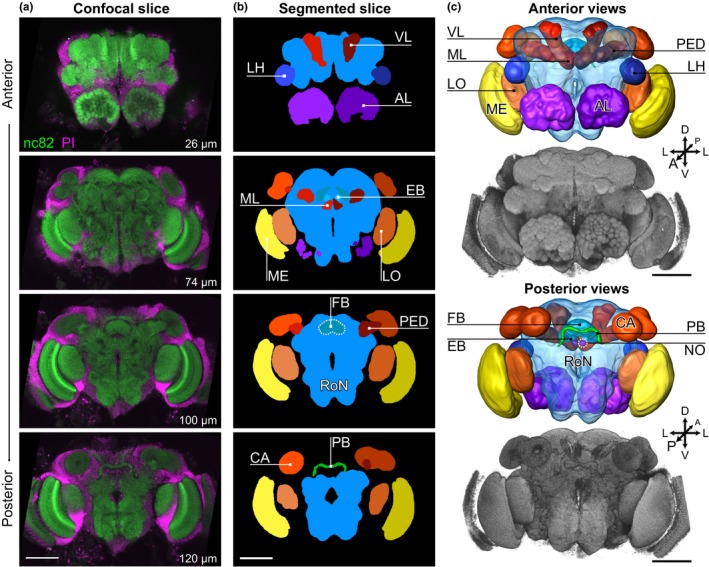
Overview of neuropils measured. Scale bars depict 100 μm in all panels. (a) Selected slices through a single *Nasonia vitripennis* brain from line L3, fluorescently labelled with nc82 (green) and PI (magenta). Bottom‐right insets indicate slice depth in μm from the anterior direction. Image contrast was increased in FIJI. (b) Schematic representation of segmented neuropils in the corresponding slices of panel A. Optic lobes (OL) consisting of lobula (LO) and medulla (ME); mushroom body (MB), consisting of the calyx (CA), pedunculus (PED), vertical lobe (VL) and medial lobe (ML). PED, VL and ML were segmented as one label, the ventral mushroom body (MB‐V); central complex (CX), consisting of the upper part of the central body (CBU, also known as fan‐shaped body), the lower part of the central body (CBL, also known as the ellipsoid body), protocerebral bridge (PB) and noduli (NO); lateral horn (LH); antennal lobe (AL) (the AL hub and glomeruli were segmented as a whole); and the remainder of the neuropil (RoN). The lamina, visible in panel A and the volume renderings of panel c, was not segmented. (c) Anterior and posterior views of a surface model based on the segmentations shown in panel b, accompanied by a volume rendering of the nc82 channel shown in panel A (using the SurfaceGen and Voltex modules, respectively, of Amira). Orientation in panel C refers to the body axis (Haverkamp & Smid, 2014). Lettering as in panel b

In the analysis of neuropil composition, 3 of 12 brains from each replicate line were analysed, resulting in pooled data sets of 9 brains per selection regime (Figure 3).

**Figure 4:**
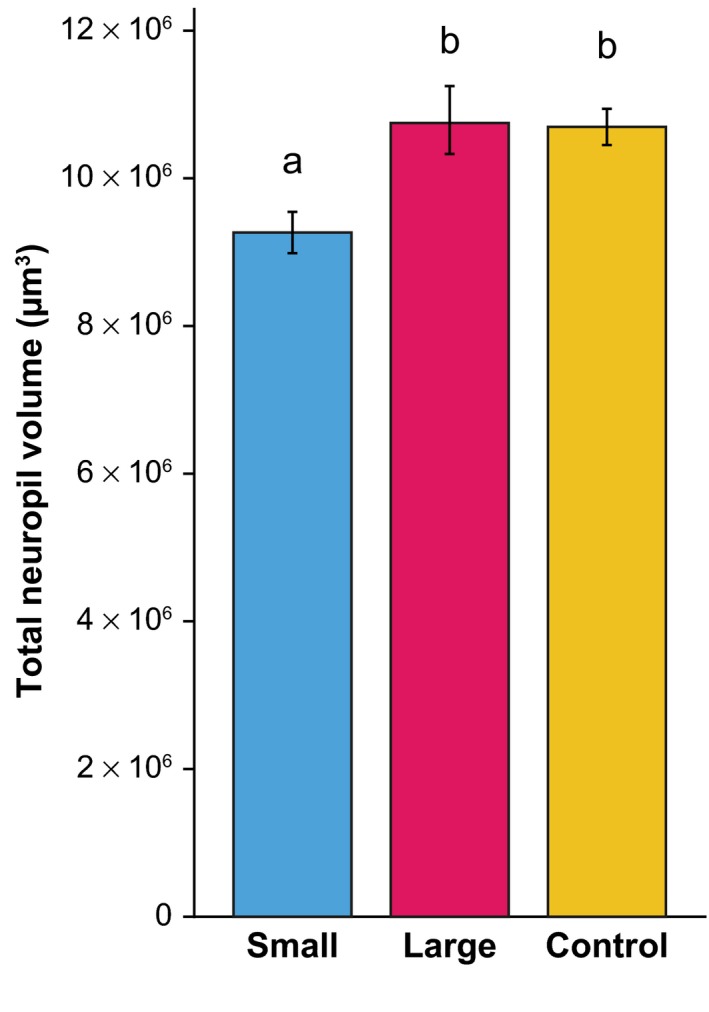
Absolute volumes of the total neuropil. Bars depict mean volume ± SE in μm^3^, *n *=* *9 for each selection regime. Letters indicate significant differences between selection regimes based on post hoc pairwise comparisons (α=0.05)

First, we analysed the absolute volume of the neuropil in the selected lines. Selection regime had a significant effect on total neuropil volume (Figure 4; $\chi_{2}^{2}$  = 8.0793, *p *=* *0.0176). Post hoc pairwise comparisons revealed that neuropil volume of wasps of the S lines was smaller (9.27 × 10^6^ ± 0.28 × 10^6^ μm^3^, M ± SE) than wasps of the C lines (10.70 × 10^6^ ± 0.25 × 10^6^ μm^3^, $\chi_{1}^{2}$ **=* *5.8393, *p *=* *0.016) and the L lines (10.75 × 10^6^ ± 0.46 × 10^6^ μm^3^, $\chi_{1}^{2}$ **=* *6.2720, *p *=* *0.012). There was no difference between the C and L lines ($\chi_{1}^{2}$ **=* *0.0077, *p *=* *0.929). On average, the total neuropil of the L lines was 16% larger than in the S lines.

**Figure 5:**
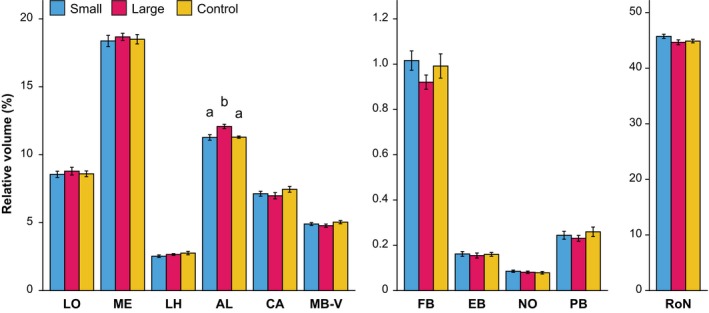
Relative volumes (mean ± SE) of the neuropils defined in Figure 3, *n *= 9 for each selection regime. Y‐axes have been split to better visualize differences between selection regimes for relatively smaller neuropils. Effects of selection regimes were first tested with a LMM, with Holm–Bonferroni correction for multiple comparisons (*m *=* *11 neuropil regions). Letters indicate significant differences between selection lines based on post hoc pairwise comparisons; unmarked bars indicate no significant effect was found for these neuropils

We further analysed the brains by comparing relative volumes of 11 neuropil regions, determined as percentages of the total neuropil volume (Figure 5). The only neuropil region that showed a significant effect of selection regime was the antennal lobe ($\chi_{2}^{2}$ **=* *19.237, Holm–Bonferroni corrected *p *=* *0.0007). Post hoc comparison revealed that the relative neuropil volume was higher in the L lines (12.08 ± 0.16%, mean ± SE) compared to the C (11.29 ± 0.08%, $\chi_{1}^{2}$ **=* *14.0360, *p *=* *0.00018) and the S (11.27 ± 0.20%, $\chi_{1}^{2}$ **=* *14.8094, *p *=* *0.00012) lines. There were no differences between the control and small lines ($\chi_{1}^{2}$ **=* *0.0104, *p *=* *0.918). Relative volumes and statistical comparisons of other neuropils are presented in Table S3.

### 3.3 Memory retention

Memory retention was analysed in 2502 wasps of the L line, 2759 wasps of the S line and 2883 wasps of the C line. Memory retention 1 day after conditioning was analysed in 12 reciprocal groups of each replicate line, resulting in 36 reciprocal groups per selection regime. Due to mortality, this number decreased over the subsequent days, resulting in a final 23 reciprocal groups per selection regime at 3 days after conditioning and 20 reciprocal groups at 5 days after conditioning.

**Figure 6:**
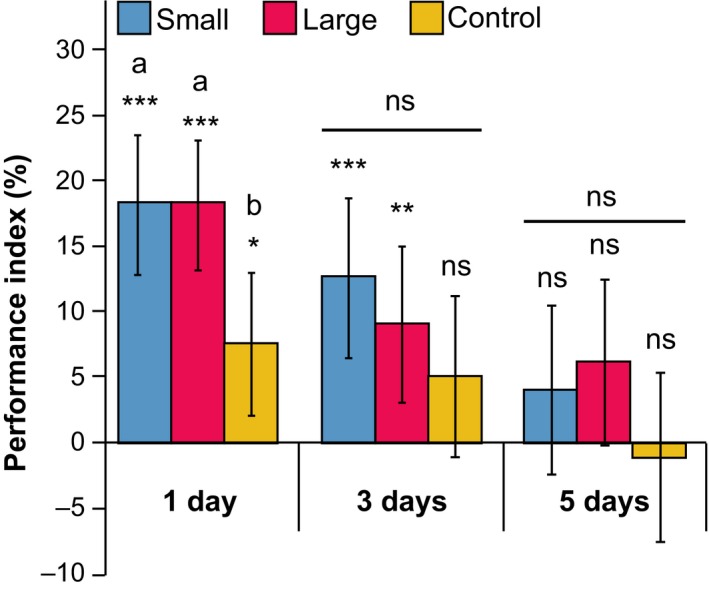
Memory retention over time for selection and control lines. Performance index (PI ± SE) shows difference in percentage of preference between reciprocally trained groups. Asterisks indicate significant memory retention (chi‐square pairwise comparisons of GLMM response); *, *p *<* *0.05; **, *p *<* *0.01; ***, *p *<* *0.001; ns, not significant; letters indicate significant differences between selection lines

Figure 6 shows memory retention (expressed as performance index, PI) levels for the different lines. There was significant memory retention ($\chi_{1}^{2}$ **=* *62.238, *p *<* *0.001), and this retention decreased over time ($\chi_{2}^{2}$ **=* *20.349, *p *<* *0.001). There was an overall difference in memory retention between the different selection regimes ($\chi_{2}^{2}$ **=* *10.971, *p *=* *0.004). Memory retention did not differ between S and L ($\chi_{1}^{2}$ **=* *0.066, *p *=* *0.796), but both lines differ in memory retention levels from C (L: $\chi_{1}^{2}$ **=* *9.002, *p *=* *0.003; S: $\chi_{1}^{2}$ **=* *7.884, *p *=* *0.005). The selected lines maintained memory up to 3 days after conditioning, and the C lines maintained memory up to 1 day after conditioning. However, there were no significant differences in decrease of memory retention level over time between the different lines ($\chi_{4}^{2}$ **=* *2.794, *p *=* *0.593). There was no difference in response rate between wasps of the different lines ($\chi_{2}^{2}$ = 1.054, *p *=* *0.591).

### 3.4 Longevity

**Figure 7:**
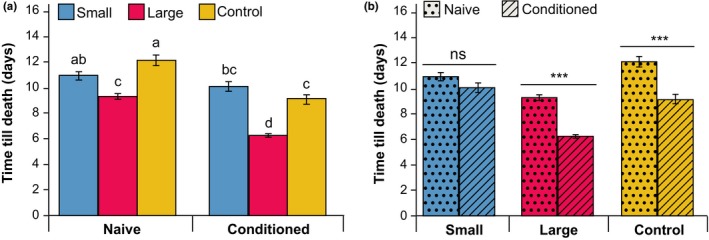
Survival of selection lines (mean ± SE), using a starting population of 180 wasps per group (60 per replicate line), with and without experiencing a single olfactory conditioning procedure. (a) Wasps with a relatively large brain have lower longevity than wasps with relatively small brains. Longevity is not improved by having a relatively small brain compared to the control lines. (b) A single olfactory conditioning experience affects longevity of wasps with a relatively large brain, but not of wasps with a relatively small brain. Asterisks and letters indicate significant differences between the groups based on Tukey HSD (see Table S5); ***, *p *<* *0.001; ns, not significant

Longevity (Figure 7) was affected by selection regime (*F*_2,1074_ = 50.433, *p *<* *0.001), experience of a conditioning trial (*F*_1,1074_ = 76.400, *p *<* *0.001), and the interaction between selection regime and conditioning was significant (*F*_2,1074_ = 7.435, *p *<* *0.001). Longevity was lower in L than in S (Tukey HSD *p *<* *0.001; Table S5) and C (Tukey HSD *p *<* *0.001). There was no difference in longevity between S and C (Tukey HSD *p *=* *0.924). Experience of a conditioning trial resulted in decreased longevity compared to naive wasps in L (Tukey HSD *p *<* *0.001) and C (Tukey HSD *p *<* *0.001), but not in S lines (Tukey HSD *p *=* *0.404).

## 4 DISCUSSION

Our bidirectional selection regime on *N. vitripennis* wasps resulted in a robust response in our proxy for relative brain size. The selection response was not sensitive to relaxation for several generations, with the difference in head–body size ratio between wasps of the L and S lines being, on average, 6.4%. Analysis of total neuropil volume showed a difference of 16% between the wasps of the L and the S lines, whereas the average body size of the L lines was smaller than the S lines. Thus, our S and L lines differed both in absolute and in relative brain volume. The response to selection, expressed as realized heritability, was lower in our study than in previous artificial selection experiments in guppies (Kotrschal et al., 2013) (i.e. 0.07 in our study and 0.48 for guppies). The regulation of relative brain size may be more complex in *N. vitripennis* than in guppies, where a change in the expression of a single gene determines relative brain size (Chen et al., 2015). The slow, but substantial selection response indicates that there is heritable genetic variation in brain size in *N. vitripennis*, but that there are constraining factors that limit the response to artificial selection. These constraints may be particularly strong due to the small size of the wasps, which causes metabolic and cognitive trade‐offs to have a large impact on the functioning of their miniaturized brains. The high metabolic costs of brain tissue (Aiello & Wheeler, 1995) may limit the development of relatively larger brains, whereas cognitive or behavioural costs may limit the formation of relatively smaller brains. Hence, relative brain size may be constrained by energetic costs on the upper limit and by functional requirements on the lower limit. Our study revealed such a cost of having relatively large brains on longevity in the selected lines (Figure 7a), but no functional benefits for olfactory memory performance (Figure 6).

### 4.1 Deviation from Haller's rule

Our previous study on phenotypic plasticity showed that brain–body size scales allometrically within the size range of the wasps of the current study, so larger wasps had absolutely larger, but relatively smaller brains (Groothuis & Smid, 2017). In contrast, the wasps of the L lines that resulted from our selection regime had larger absolute brain size but smaller body lengths than wasps of the S lines. Thus, brain–body size scaling in our selection lines occurs in opposite direction of brain–body size scaling induced by phenotypic plasticity. This is in line with Figure 2, which shows the regression of body length and head width of the S, L and C lines after the selection process; the three lines differed in intercept, with L above C and C above S. This suggests that grade shifts have occurred, which are elevation displacements that illustrate a difference in the level of encephalization at similar body sizes between different groups (Eberhard & Wcislo, 2011; Striedter, 2005). The genes under selection are therefore likely involved in phenotypic plasticity of brain–body size scaling.

Our finding bears comparison with a recent analysis of brain scaling in 40 cichlid species (Tsuboi et al., 2016). Plotting both the inter‐ and intraspecific allometric brain–body size relationships showed that the variation in intraspecific intercepts, rather than in the slopes, explained variation in relative brain size across species within a family (Tsuboi et al., 2016). Thus, the variation in relative brain size between these cichlid species was explained by overall differences in encephalization level, and not by species‐specific variation in brain–body size scaling dynamics. Our results support this view and indicate that there was genetic variation in encephalization level in the starting (HVRx) population. This type of genetic variation may underlie evolution of differences in relative brain size.

### 4.2 Brain morphology

Our neuropil analysis (Figure 5) shows that our selection regime selectively affected the relative volume of the antennal lobe, which was larger in the L lines than in the S and C lines. These results are different from our previous work on body size effects on brain scaling and brain morphology in *N. vitripennis*, where we found differences in several neuropils, but not the AL (Groothuis & Smid, 2017). However, in that previous study, we induced phenotypic plasticity in brain and body size, using varying degrees of scramble competition in an isogenic line. Genetic variation in brain size and phenotypic plasticity in brain size therefore appear to have different effects on neuropil composition, which implies that different mechanisms may be involved in regulating neuropil plasticity. Moreover, the difference in absolute neuropil volumes was much larger in our previous study addressing phenotypic plasticity: approximately 152% (Groothuis & Smid, 2017) in contrast to 16% in the present study (Figure 4).

These results suggest that the antennal lobe may have a fixed relative volume under isogenic scramble competition but a variable relative volume when genetic variation is present, whereas the opposite is the case for the other neuropils. In comparison, both bumblebees and honeybees (which have limited genetic variation in the same colony, but two‐ to three‐fold variation in brain volume), relative AL volume does not vary over the size range of these species (Mares, Ash, & Gronenberg, 2005). Such constant scaling of AL volume was confirmed for honeybees in a later study (Gronenberg & Couvillon, 2010). By contrast, scramble competition in an isogenic strain of *T. evanescens* resulted in relatively smaller AL glomeruli in smaller brains (van der Woude & Smid, 2016). Thus, the relation between relative neuropil volume, body size and genetic background deserves further study.

### 4.3 Memory retention

In our study, there was no effect of relative brain size on memory performance. Wasps of the L and S lines showed similar levels and duration of memory retention. In contrast, a positive effect of larger brains on memory retention levels was recorded in our previous study on phenotypic plasticity in absolute brain size in *N. vitripennis* (Van der Woude et al., 2018). Furthermore, a study on guppies recorded higher memory retention levels in guppies that were selected for relatively larger brains (Kotrschal et al., 2013). Though other measures of brain size were used, thus hampering a comparison between guppies and wasps, the 16% difference in neuropil volume between *N. vitripennis* wasps of the L and S lines in our study exceeds the 9% difference in brain weight recorded in guppies, even without accounting for the aforementioned decrease in body size of the L lines. Hence, the similarity in olfactory memory performance of our selected *N. vitripennis* lines was surprising, but in line with our findings on relative volumes of the mushroom bodies, which are important structures in the insect brain that are involved in learning and memory formation (Perry & Barron, 2012). Indeed, our previous study on phenotypic plasticity in body size showed that wasps with brains that were larger in absolute volume had higher memory retention levels (Van der Woude et al., 2018), and relatively larger mushroom bodies (Groothuis & Smid, 2017). In the current study, there was no difference in relative volumes of the mushroom bodies between the S, C and L lines (Figure 5), which is in line with the observed similarity in olfactory memory performance between wasps of the S and L lines. The combined results of the memory performance tests and neuropil analyses suggest that the costs and benefits of genetic changes in relative brain size may not be related to memory but to olfaction. The larger AL volume could have had an effect on olfactory sensitivity, which was not analysed in our study. Such a possible effect was, however, not reflected in improved olfactory memory in the L lines. A recent study in *Drosophila*, where fly larva reared under crowded conditions yielded adults with reduced AL volume, also reported no effects on olfactory memory (Wang, Amei, de Belle, & Roberts, 2018). Interestingly, a recent study on artificial selection on visual learning, using the same strain of *N. vitripennis* that was used in our study*,* showed a rapid effect of selection on memory performance, but no effect on relative volume of the mushroom bodies, the antennal lobes or any of the other investigated neuropils (Liefting, Hoedjes, Le Lann, Smid, & Ellers, 2018). This confirms that mechanisms underlying phenotypic variation in relative mushroom body and/or brain size and the correlations with memory performance may be different from those involved in genetic variation.

Our study also revealed a significantly higher level of memory retention abilities in the selected (S and L) than in the unselected C lines. Memory in the unselected C lines is, however, similar as in the original starting population HVRx (Figure S4). This indicates that our bidirectional selection regime resulted in increased memory retention abilities, whereas memory retention abilities remained unchanged in the C lines. Our neuropil analysis suggests that this observed increase in both S and L lines does not have a basis in mushroom body volume, but potentially in other aspects of brain morphology not recorded in the present study. Interestingly, previous comparisons of the effect size of conditioning on different strains of *N. vitripennis* showed that the HVRx strain has a lower effect size, reflected in lower memory retention levels than the isogenic AcymCx strain (Koppik, Hoffmeister, Brunkhorst, Kieß, & Thiel, 2015). This effect is also visible in our control experiments (Figure S4). It is intriguing that our selection regime in both directions selected for improved memory retention abilities, but the underlying cause of this effect remains elusive.

### 4.4 Longevity

Wasps with relatively larger brains live shorter than wasps with relatively small brains (Figure 7a). This illustrates the constitutive, global costs of brain tissue, in line with the theory that brain tissue is metabolically expensive (Aiello & Wheeler, 1995; Snell‐Rood, Papaj, & Gronenberg, 2009). The effect on longevity may, however, also be partly induced by the smaller body size of the L lines. Smaller wasps are known to suffer from reduced longevity (Sykes, Innocent, Pen, Shuker, & West, 2007), albeit that the size differences between our selected lines are rather small. Our results also show that C and L lines, but not the S lines, had reduced longevity after an olfactory conditioning experience with a host as a reward, which is known to induce long‐term, protein synthesis‐dependent memory (Hoedjes, Kralemann, van Vugt, Vet, & Smid, 2014). (Figure 7b). Memory formation can affect neuropil size and relative neuropil distribution. For instance, the relative volume of the mushroom bodies was found to increase with host‐finding experience in the butterfly *Pieris rapae* (Snell‐Rood et al., 2009). Such experience‐dependent plasticity, in combination with the associated changes in metabolic costs, constitutes the induced costs of learning (Snell‐Rood et al., 2009). This could also underlie the learning‐induced costs that were found in *Drosophila*, which live shorter after forming long‐term memory (Mery & Kawecki, 2005) or when selected for improved aversion learning (Burger, Kolss, Pont, & Kawecki, 2008). It should be noted that *N. vitripennis* wasps do not actually oviposit within the single hour of the conditioning experience, but feed on their host after drilling (Hoedjes, Kralemann, et al., 2014), and this host feeding induces egg maturation (Whiting, 1967). Thus, the costs that underlie the decreased longevity after a conditioning experience in the L wasps may be caused by the host encounter, the host feeding‐induced egg maturation and long‐term memory formation, but not oviposition. That a conditioning experience did affect longevity of wasps of the L and C lines but not the S lines in our study, suggests weaker energetic constraints in wasps with relatively small brains, possibly due to reallocation of resources from processes involved in operating and maintaining the smaller brain to general somatic and reproductive processes. Thus, the wasps of the S lines benefit from having a relatively smaller brain by reduced sensitivity to the negative effects of conditioning on longevity.

## 5 CONCLUSION

In the ongoing investigation of the question whether and how bigger brains are better (Chittka & Niven, 2009), we have provided a comprehensive and important dataset from the perspective of the smallest animal species studied in this regard, showing that bigger brains are not necessarily better, but certainly more costly. We show that our selection approach is feasible and can provide novel insights into the costs and gains of genetic variation in brain size. Future studies may include additional model species and the effects of genetic variation in relative brain size on phenotypic plasticity of brain morphology and behaviour.

## AUTHOR CONTRIBUTIONS

EW, JG and HMS designed the study and interpreted the data. EW and JG performed experiments, analysed data and prepared the manuscript. HMS coordinated the study and helped drafting the manuscript.
