# Supplementary material for: No gains for bigger brains: Functional and neuroanatomical consequences of relative brain size in a parasitic wasp
Source: J Evol Biol. 2019 Apr 13;32(7):694–705. doi: 10.1111/jeb.13450 (PMC6850633; doi:10.1111/jeb.13450)
Supplement: Supplementary file 1 [file JEB-32-694-s001.docx]

**Supplementary Results**

*S1 Response to selection*

**Table S1. Measured head and body size parameters for wasps of selected lines.** Average body length in µm (±SE), head width in µm (±SE) and ratio between head width and body length (±SE) per generation in the selected lines. Final column shows the difference in average head-body ratio between the L and S lines (L-S) in percentages. N=150 for each cell except generation 0, in which N=300.

|  | Body length | | | Head width | | | Head - body ratio | | | |
| --- | --- | --- | --- | --- | --- | --- | --- | --- | --- | --- |
| Generation | Small | Large | Control | Small | Large | Control | Small | Large | Control | Difference ratio L - S (%) |
| 0 | 1967 ± 10.2 | 1967 ± 10.2 |  | 668 ± 3.3 | 668 ± 3.3 |  | 0.3403 ± 0.0011 | 0.3403 ± 0.0011 |  |  |
| 1 | 2426 ± 7.5 | 2350 ± 11.0 |  | 773 ± 2.1 | 776 ± 3.1 |  | 0.3186 ± 0.0005 | 0.3306 ± 0.0008 |  | 3.75 |
| 2 | 2240 ± 10.1 | 2297 ± 12.2 |  | 740 ± 2.6 | 738 ± 3.4 |  | 0.3307 ± 0.0010 | 0.3215 ± 0.0007 |  | -2.78 |
| 3 | 2230 ± 10.2 | 2280 ± 10.1 |  | 734 ± 3.5 | 756 ± 2.9 |  | 0.3295 ± 0.0007 | 0.3317 ± 0.0008 |  | 0.68 |
| 4 | 2315 ± 9.6 | 2323 ± 9.8 |  | 746 ± 2.7 | 748 ± 2.7 |  | 0.3225 ± 0.0006 | 0.3221 ± 0.0007 |  | -0.14 |
| 5 | 2271 ± 10.9 | 2245 ± 10.3 |  | 725 ± 3.2 | 732 ± 3.0 |  | 0.3194 ± 0.0006 | 0.3262 ± 0.0006 |  | 2.15 |
| 6 | 2299 ± 14.6 | 2298 ± 12.3 |  | 734 ± 4.7 | 749 ± 3.6 |  | 0.3195 ± 0.0007 | 0.3262 ± 0.0010 |  | 2.10 |
| 7 | 2272 ± 11.3 | 2317 ± 11.1 |  | 726 ± 3.3 | 746 ± 3.2 |  | 0.3196 ± 0.0007 | 0.3223 ± 0.0009 |  | 0.84 |
| 8 | 2239 ± 12.6 | 2256 ± 10.7 |  | 729 ± 3.6 | 735 ± 3.2 |  | 0.3259 ± 0.0007 | 0.3260 ± 0.0009 |  | 0.04 |
| 9 | 2208 ± 7.8 | 2230 ± 8.8 |  | 719 ± 2.4 | 727 ± 2.7 |  | 0.3257 ± 0.0007 | 0.3262 ± 0.0008 |  | 0.16 |
| 10 | 2341 ± 8.4 | 2234 ± 13.6 |  | 741 ± 2.4 | 731 ± 3.9 |  | 0.3166 ± 0.0007 | 0.3276 ± 0.0007 |  | 3.47 |
| 11 | 2281 ± 9.0 | 2293 ± 10.0 |  | 725 ± 2.5 | 750 ± 2.7 |  | 0.3180 ± 0.0006 | 0.3276 ± 0.0006 |  | 2.99 |
| 12 | 2313 ± 12.9 | 2293 ± 12.0 |  | 724 ± 3.3 | 752 ± 3.4 |  | 0.3134 ± 0.0007 | 0.3284 ± 0.0006 |  | 4.79 |
| 13 | 2284 ± 11.8 | 2321 ± 9.2 |  | 725 ± 3.3 | 752 ± 2.4 |  | 0.3177 ± 0.0007 | 0.3242 ± 0.0011 |  | 2.06 |
| 14 | 2398 ± 6.9 | 2281 ± 10.8 |  | 751 ± 2.0 | 748 ± 2.9 |  | 0.3132 ± 0.0007 | 0.3282 ± 0.0006 |  | 4.78 |
| 15 | 2388 ± 7.7 | 2385 ± 8.4 |  | 747 ± 1.8 | 764 ± 2.1 |  | 0.3131 ± 0.0007 | 0.3209 ± 0.0009 |  | 2.47 |
| 16 | 2378 ± 10.1 | 2258 ± 10.6 |  | 737 ± 2.6 | 746 ± 3.0 |  | 0.3104 ± 0.0008 | 0.3307 ± 0.0006 |  | 6.54 |
| 17 | 2293 ± 11.4 | 2264 ± 9.2 |  | 727 ± 3.4 | 740 ± 2.9 |  | 0.3169 ± 0.0007 | 0.3268 ± 0.0006 |  | 3.12 |
| 18 | 2345 ± 10.9 | 2237 ± 9.7 |  | 736 ± 2.9 | 747 ± 2.9 |  | 0.3143 ± 0.0007 | 0.3340 ± 0.0008 |  | 6.26 |
| 19 | 2299 ± 10.2 | 2226 ± 14.5 |  | 721 ± 2.6 | 731 ± 4.2 |  | 0.3140 ± 0.0006 | 0.3286 ± 0.0006 |  | 4.65 |
| 20 | 2367 ± 8.7 | 2253 ± 10.3 |  | 737 ± 2.4 | 749 ± 2.6 |  | 0.3115 ± 0.0006 | 0.3324 ± 0.0009 |  | 6.74 |
| 21 | 2279 ± 9.5 | 2289 ± 10.4 |  | 719 ± 2.5 | 742 ± 2.9 |  | 0.3153 ± 0.0007 | 0.3244 ± 0.0007 |  | 2.88 |
| 22 | 2312 ± 8.6 | 2267 ± 9.9 |  | 724 ± 2.5 | 755 ± 2.9 |  | 0.3133 ± 0.0005 | 0.3334 ± 0.0006 |  | 6.42 |
| 23 | 2331 ± 10.8 | 2194 ± 14.3 |  | 727 ± 2.9 | 724 ± 4.3 |  | 0.3122 ± 0.0007 | 0.3301 ± 0.0006 |  | 5.75 |
| 24 | 2320 ± 11.0 | 2240 ± 14.9 |  | 722 ± 3.1 | 738 ± 4.6 |  | 0.3115 ± 0.0007 | 0.3298 ± 0.0008 |  | 5.90 |
| 25 | 2383 ± 8.2 | 2236 ± 12.5 | 2347 ± 9.1 | 743 ± 2.6 | 732 ± 3.8 | 753 ± 2.6 | 0.3118 ± 0.0006 | 0.3278 ± 0.0009 | 0.3208 ± 0.0006 | 5.14 |
| 26 | 2341 ± 10.2 | 2252 ± 10.6 | 2266 ± 13.3 | 728 ± 2.9 | 745 ± 3.1 | 725 ± 4.1 | 0.3114 ± 0.0007 | 0.3310 ± 0.0006 | 0.3202 ± 0.0008 | 6.30 |
| 27 | 2341 ± 12.0 | 2286 ± 10.4 | 2384 ± 9.7 | 733 ± 3.3 | 751 ± 3.2 | 763 ± 3.0 | 0.3134 ± 0.0007 | 0.3286 ± 0.0006 | 0.3199 ± 0.0006 | 4.86 |
| 28 | 2329 ± 9.2 | 2220 ± 12.6 | 2218 ± 11.2 | 718 ± 2.4 | 733 ± 4.0 | 719 ± 3.2 | 0.3085 ± 0.0006 | 0.3302 ± 0.0006 | 0.3245 ± 0.0006 | 7.02 |
| 30 | 2289 ± 12.7 | 2108 ± 16.2 | 2237 ± 11.6 | 709 ± 3.4 | 702 ± 4.8 | 718 ± 3.5 | 0.3100 ± 0.0007 | 0.3334 ± 0.0008 | 0.3209 ± 0.0006 | 7.55 |
| 33 | 2293 ± 8.3 | 2200 ± 11.7 | 2251 ± 10.3 | 720 ± 2.5 | 737 ± 3.3 | 731 ± 2.9 | 0.3142 ± 0.0007 | 0.3352 ± 0.0007 | 0.3251 ± 0.0007 | 6.67 |
| 40 | 2244 ± 13.1 | 2172 ± 13.8 | 2238 ± 10.0 | 713 ± 4.0 | 732 ± 4.3 | 739 ± 3.1 | 0.3180 ± 0.0009 | 0.3371 ± 0.0008 | 0.3305 ± 0.0009 | 6.03 |

*Deviation from Haller’s rule*


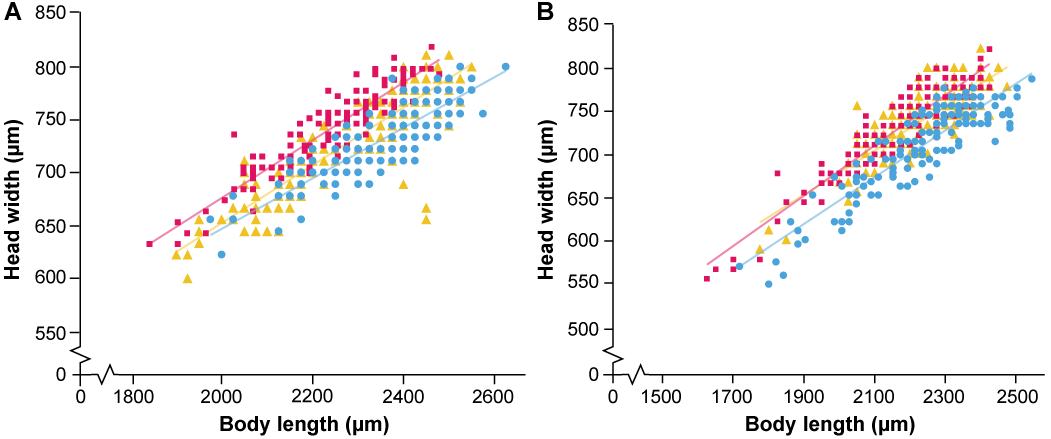


**Figure S2.** Head width and body length of wasps selected for relatively large (magenta squares) and small (blue circles) head-body ratio, and unselected control lines (yellow triangles). Measurements are shown for (**A**) generation 26 and (**B**) generation 40. Regression analysis was performed on mean-centered body lengths, because differences in head-body ratio can then be revealed by differences in the intercepts.

Statistical analysis was performed on mean-centered body lengths (subtraction of mean body length of whole generation), whereas Figure S2 shows uncorrected body lengths and head widths. Using mean-centered body lengths for analysis ensured that differences in head-body ratio between the selected lines are reflected by differences in the intercept.

Regression with mean-centered body length in generation 26: significant differences in intercept (F_2,444_=48.523, *p*<0.001), but not in slope (F_2,444_=2.844, *p*=0.059). R^2^=0.805.

Large: HW=0.273×sBL + 757.123

Small: HW=0.238×sBL + 717.817

Control: HW=0.273×sBL + 733.394

Regression with mean-centered body length in generation 33: significant differences in intercept (F_2,444_=36.466, *p*<0.001), but not in slope (F_2,444_=0.670, *p*=0.512). R^2^=0.784.

Large: HW=0.260×sBL + 749.048

Small: HW=0.244×sBL + 709.134

Control: HW=0.245×sBL + 730.396

Regression with mean-centered body length in generation 40: significant differences in intercept (F_2,444_=60.432, *p*<0.001), but not in slope (F_2,444_=2.042, *p*=0.131). R^2^=0.809.

Large: HW=0.292×sBL + 745.181

Small: HW=0.271×sBL + 705.932

Control: Hw=0.258×sBL + 734.108

*Brain morphology data*

**Table S3**

Excel file

*S4 Memory retention levels*

One day after conditioning, wasps from the S lines showed a mean PI (± SE) of 18.37 ± 5.39%, L of 18.36 ± 5.01% and C of 7.62 ± 5.51%. This memory retention was significant in all lines (S: χ^2^_1_ = 28.878, *p*<0.001; L: χ^2^_1_ = 34.082, *p*<0.001; C: χ^2^_1_ =5.096, *p*=0.024). There was a significant difference in the level of memory retention between the selected and C lines 1 day after conditioning (S vs. C: χ^2^_1_ = 5.26, *p*=0.022; L vs. C: χ^2^_1_ = 7.84, *p*=0.005), but not between the S and L lines (χ^2^_1_ = 0.33, *p*=0.567). Three days after conditioning, wasps from the S lines showed a PI of 12.73 ± 6.17%, L of 9.14 ± 6.03% and C of 5.14 ± 6.22%. This memory retention was significant in the S and L lines (S: χ^2^_1_ = 13.935, *p*<0.001; L: χ^2^_1_ = 7.429, *p*=0.006), but not in C (χ^2^_1_ =2.363, *p*=0.124). There were no significant differences in the level of memory retention between the lines 3 days after conditioning (S vs. C: χ^2^_1_ = 2.57, *p*=0.109; L vs. C: χ^2^_1_ = 0.82, *p*=0.365; S vs. L: χ^2^_1_ = 0.46, *p*=0.498). Five days after conditioning, S showed a PI of 4.10 ± 6.52%, L of 6.23 ± 6.40% and C of -1.09 ± 6.52%. None of this was significant memory retention (S: χ^2^_1_ = 1.20, *p*=0.273; L: χ^2^_1_ = 2.783, p=0.095; C: χ^2^_1_ =0.084, *p*=0.772), and there were no differences in memory retention levels between the lines (S vs. C: χ^2^_1_ = 0.95, *p*=0.329; L vs. C: χ^2^_1_ = 1.96, *p*=0.161; S vs. L: χ^2^_1_ = 0.21, *p*=0.649).

Response rate was defined as the percentage of wasps that made a choice, out of the total amount of wasps that were inserted into the T-maze. There was no difference in response rate between wasps of the different lines (χ^2^_2_ = 1.054, *p*=0.591). Time after conditioning did affect response rate (χ^2^_2_ = 33.296, *p*<0.001), with higher response rates longer after conditioning (day 1 – 3: χ^2^_1_ = 11.363, *p*<0.001; day 3 – 5: χ^2^_1_ = 5.742, *p*=0.017; day 1 – 5: χ^2^_1_ = 31.834, *p*<0.001). The average response rate (±SE) was 72.53 ± 0.24 % on day 1, 77.79 ± 0.19 % on day 3 and 81.21 ± 0.27 % on day 5. There was no significant effect of the interaction between the lines and time after conditioning (χ^2^_4_ = 1.302, *p*=0.861) on response rate.

*Memory comparison with HVRx and AsymCx strains*

We performed additional controls to compare memory performance of our selection and control lines to memory performance of the HVRx starting population and the AsymCx strain that we used in our previous study (Van der Woude and Smid 2017). We therefore analyzed memory retention of 2470 HVRx and 2179 AsymCx wasps following the same methodology as for our selection and control lines (Figure S4). There was significant memory retention (GLMM: conditioning χ^2^_1_=157.37, *p*<0.001), and this retention decreased over time (GLMM: conditioning*time χ^2^_2_=32.59, *p*<0.001). There was an overall difference in memory retention between the different lines (GLMM: conditioning*line χ^2^_4_=67.64, *p*<0.001). Memory retention did not differ between S and L (χ^2^_1_=0.090, *p*=0.767), nor between C and HVRx. (χ^2^_1_=0.840, *p*=0.359). All other pairwise comparisons did yield significant differences (AsymCx – L: χ^2^_1_=18.46, *p*<0.001; AsymCx – C: χ^2^_1_=61.04, *p*<0.001; AsymCx – S: χ^2^_1_=44.81, *p*<0.001; AsymCx – HVRx: χ^2^_1_=22.23, *p*<0.001; L – C: χ^2^_1_=11.88, *p*<0.001; L – HVRx: χ^2^_1_=5.97, *p*=0.015; C – S: χ^2^_1_=10.64, *p*=0.001; HVRx – S: χ^2^_1_=4.95, *p*=0.026). Memory was maintained up to 3 days after conditioning in HVRx, and up to 5 days in AsymCx.


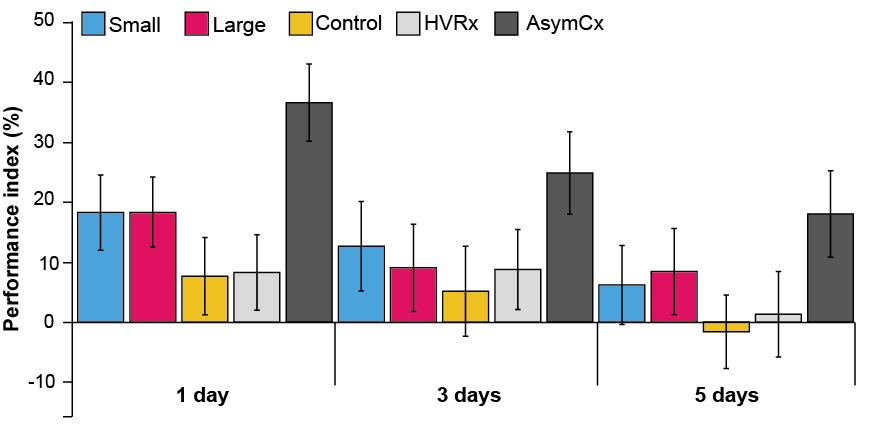


**Figure S4.** Memory retention over time for selection and control lines, and additional controls with the HVRx starting population and isogenic AsymCx line. Performance index (mean ± SE) shows difference in percentage of preference between reciprocally trained groups.

*S5 Longevity*

Within the wasps that received a conditioning trial, mean longevity (± SE) was 10.11 ± 0.38 days in S, 6.28 ± 0.13 days in L and 9.17 ± 0.37 days in C. Within naive wasps, mean longevity was 10.98 ± 0.32 days in S, 9.32 ± 0.22 days in L and 12.17 ± 0.42 days in C.

**Table S5.** All TukeyHSD comparisons of longevity in naive and conditioned wasps of the three lines. These values were used for Figure 7A.

|  |  | Naive | | | Conditioned | | |
| --- | --- | --- | --- | --- | --- | --- | --- |
|  |  | Small | Large | Control | Small | Large | Control |
| Naive | Small | - | - | - | - | - | - |
|  | Large | *p*=0.004 | - | - | - | - | - |
|  | Control | *p*=0.097 | *p*<0.001 | - | - | - | - |
| Conditioned | Small | *p*=0.404 | *p*=0.513 | *p*<0.001 | - | - | - |
|  | Large | *p*<0.001 | *p*<0.001 | *p*<0.001 | *p*<0.001 | - | - |
|  | Control | p=0.001 | *p*=0.999 | *p*<0.001 | *p*=0.304 | *p*<0.001 | - |

**Supplementary References**

Van der Woude, E., Huigens, M. E. & Smid, H. M. 2018. Differential effects of brain size on memory performance in parasitic wasps. *J. Anim. Behav*. **141**: 57-66
